# Publishing online identification keys in the form of scholarly papers

**DOI:** 10.3897/zookeys.205.3581

**Published:** 2012-07-04

**Authors:** Lyubomir Penev, Pierfilippo Cerretti, Hans-Peter Tschorsnig, Massimo Lopresti, Filippo Di Giovanni, Teodor Georgiev, Pavel Stoev, Terry L. Erwin

**Affiliations:** 1 Pensoft Publishers, Sofia, Bulgaria; 2 Institute of Biodiversity and Ecosystem Research, Bulgarian Academy of Sciences, Sofia; 3 Dipartimento di Biologia e Biotecnologie “Charles Darwin”, Università di Roma “La Sapienza”, Piazzale A. Moro 5, 00185, Rome, Italy; 4 Centro Nazionale per lo Studio e la Conservazione della Biodiversità Forestale – Corpo Forestale dello Stato, Via Carlo Ederle 16/A, 37100, Verona, Italy; 5 Staatliches Museum für Naturkunde, Rosenstein 1, 70191 Stuttgart, Germany; 6 National Museum of Natural History, Sofia, Bulgaria; 7 Department of Entomology, MRC 187, National Museum of Natural History, Smithsonian Institution, P. O. Box 37012, Washington, DC 20013-7012 USA

One of the main deficiencies in publishing and dissemination of online interactive identification keys produced through various software packages, such as DELTA, Lucid, MX and others, is the lack of a permanent scientific record and a proper citation mechanism of these keys. In two earlier papers, we have discussed some models for publishing raw data underpinning interactive keys (Penev et al. 2009; Sharkey et al. 2009). Here we propose a method to incentive authors of online keys to publishing these through the already established model of “Data Paper” (Chavan and Penev 2011, examples: Narwade et al. 2011, Van Landuyt et al. 2012, Schindel et al. 2011, Pierrat et al. 2012, see also Pensoft's Data Publishing Policies and Guidelines). For clarity, we propose a new article type for this format, “Online Identification Key”, to distinguish it from the “Data Paper” in the narrow sense.

The model is demonstrated through an exemplar paper of Cerretti et al. (2012) in the current issue of ZooKeys. The paper describes the main features of an interactive key to the Palaearctic genera of the family Tachinidae (Diptera) implemented as an original web application. The authors discuss briefly the advantages of these tools for both taxonomists and general users, and point out the need of shared, standardized protocols for taxa descriptions to keep matrix-based interactive keys easily and timely updated.

The format of the “Online Identification Key” paper largely resembles the structure of Data Papers proposed by Chavan and Penev (2011) on the basis of the Ecological Metadata Language (EML) and developed further in Pensoft's Data Publishing Policies and Guidelines. An “Online Identification Key” paper should focus on a formal description of the technical details and content of an online key that is what is often called “metadata”. For example, an “Online Identification Key” paper has a title, author(s), abstract and keywords like any other scientific paper; it should also include in the first place: the URL of an open access version of the online key and possibly also the data underpinning the key, information on the history of and participants in the project, the software used and its technical advantages and constraints, licenses for use, taxonomic and geographic coverage, lists and descriptions of the morphological characters used, and literature references.

In contrast to conventional data papers, the “Online Identification Key” papers do not require compulsory publication of raw data files underpinning a key, although such a practice is highly recommended and encouraged. There might be several obstacles in publishing raw data that can be due to copyright issues on either data or source codes. It is mandatory, however, for the online keys published in this way to be freely available for use to anyone, by just clicking the URL address published in the paper.

The publication of an online key in the form of a scholarly article is a pragmatic compromise between the dynamic structure of the internet and the static character of scientific articles. The author(s) of the key will be able to continuously update the product, to the benefit of its users. At the same time, the users will have available a citation mechanism for the online key, identical to that used for any other scientific article, to properly credit the authors of the key.
